# Novel Models for Assessing and Pathophysiology of Hepatic Ischemia–Reperfusion Injury Mechanisms

**DOI:** 10.3390/medicina60091507

**Published:** 2024-09-15

**Authors:** Connor Whalen, Arun Verma, Kento Kurashima, Jasmine Carter, Hala Nazzal, Ajay Jain

**Affiliations:** Department of Pediatrics, St. Louis University School of Medicine, St. Louis, MO 63104, USA; arun.verma@health.slu.edu (A.V.); kento.kurashima@health.slu.edu (K.K.); jasminej.carter2020@gmail.com (J.C.); hnazzal@micds.org (H.N.); ajay.jain@slucare.ssmhealth.com (A.J.)

**Keywords:** ischemia–reperfusion injury, reactive oxygen species, murine, porcine, cell line, machine perfusion

## Abstract

Hepatic ischemia–reperfusion injury (IRI) is a major cause of postoperative hepatic dysfunction and liver failure involving cellular damage to previously ischemic tissues to which blood flow is restored. The reestablishment of blood flow is essential for salvaging ischemic tissues. The reperfusion itself, however, can paradoxically lead to further cellular damage, which involves a multi-factorial process resulting in extensive tissue damage, which can threaten the function and viability of the liver and other organ systems. The following review outlines multiple models for in-lab analysis of the various hepatic IRI mechanisms, including murine, porcine, cell lines, and machine perfusion models.

## 1. Introduction

The different surgical options necessary for treating complications in the liver, ranging from liver failure to intrahepatic lesions, require a period of ischemia. Ischemia–reperfusion injury (IRI) involves two interacting phases: local ischemia injury and inflammation-mediating reperfusion injury [1]. There are two distinct types of hepatic IRI: warm IRI and cold IRI. These share a very similar underlying pathophysiology. The ‘warm’ IRI begins at the level of the hepatic cell and develops in situ due to liver surgery, organ procurement, and other related forms of shock or trauma. The ‘cold’ IRI is initiated by liver sinusoidal endothelial cells and is usually coupled with ‘warm’ IRI during surgery, but can also occur during the transportation of the liver [2,3]. As a major risk factor for postoperative complications, including mortality, hepatic IRI can not only lead to liver failure and dysfunction, but can also affect other remote organs and, in more severe cases, can cause multiple organ dysfunction syndrome or systemic inflammatory response syndrome, which severely affect patients’ chances of survival and their potential quality of life [4]. Since liver transplantation remains the only effective treatment for patients with terminal liver diseases, its critical obstacle of organ shortage is aggravated by hepatic IRI-related forms of organ damage [5]. A better understanding of the mechanisms involved in IRI will provide insights into improving the treatment regimen for IRI. This is critical, as effective methods for preventing or minimizing hepatic IRI during and immediately following liver surgery are urgently needed.

According to the Organ Procurement and Transplant Network, there were more than 13,500 patients with liver disease awaiting a transplant in 2019, while only about 8250 livers were available for transplantation [6]. Unfortunately, such a trend continues in transplant medicine, and, on average, eight people die every day while on the waiting list for a liver transplant [7]. Despite this great need for organs, livers from deceased donors are discarded at an alarmingly high rate. According to the Scientific Registry of Transplant Recipients for the United States, 9% of deceased donor livers are discarded [8]. Such livers include those with severe levels of steatosis, those from elderly donors, and livers subject to donation after circulatory death (DCD) [9]. These livers are frequently discarded due to the susceptibility of these organs to IRI, which results in a range of poor outcomes, from severe liver dysfunction and morbidity to primary non-function and even patient death [9]. To attempt to circumvent the acute shortages, marginal donor livers (MDLs) have seen increasing use in liver transplantation to address the significant shortage of organs. However, MDLs are also more vulnerable to IRI [9]. As mentioned, IRI in these livers can result from various types of ischemia. The purpose of this review is to outline and understand the methodologies presently being used to elucidate the mechanisms of liver IRI and to develop new therapeutic approaches, in light of recent scientific evidence.

## 2. Pathophysiology

**Figure 1:**
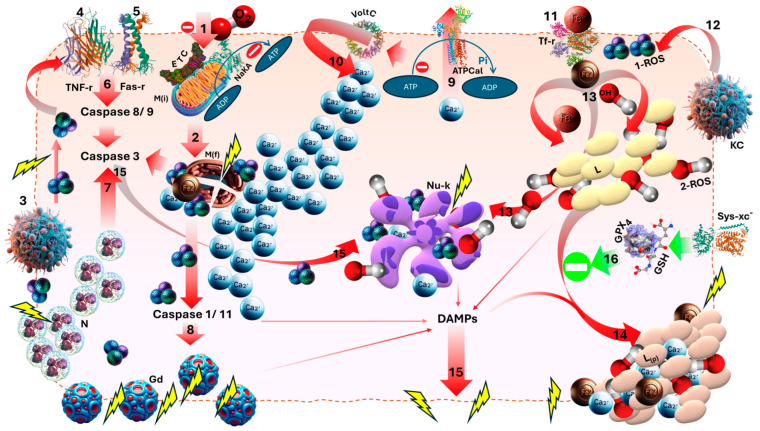
The cellular and molecular basis of hepatocellular ischemia–reperfusion injury: #1: Ischemia → anoxia → inhibits Electron Transport Chain-mediated cellular respiration and ATP generation → enzymes inhibited + pumps stop working + acidosis. #2: ATP depletion, iron overload-induced mitochondrial fragmentation, and membrane rupture releasing calcium + free iron + primary ROS shower. #3: Kupffer Cells activation → (a) generation of primary ROS, (b) activation of TNF (#4) and Fas (#5) receptor-mediated pathway triggering the Caspase cascade (#6) leading up to terminal apoptotic effector Caspase 3, (c) recruitment of neutrophils causing membrane and organelle damage and potentiation of Caspase 3 (#7). #8: Primary ROS-induced Caspase 1/11-mediated Gasdermin cleavage → Gasdermin binds to membrane lipids, creating transmembrane pores. #9: ATP depletion-induced calcium pump failure → loss of transmembrane calcium gradient → open voltage-gated calcium channels → massive calcium influx (#10) → organelle disruption and membrane rupture. This adds to calcium efflux from damaged mitochondria and other organelles. Iron is already transported intracellularly via Transferrin Receptor (#11) from ferric to ferrous state and in equilibrium with cytosolic and mitochondrial Heme, Ferritin, and iron–Sulfur Clusters. #12: Kupffer Cells, neutrophils, and mitochondria-generated primary ROS initiate and potentiate the generation of secondary ROS. #13: Fenton Reaction and Haber–Weiss Reactions converting ferrous back to ferric ions and generating the most reactive ROS: the hydroxyl radical—the terminal effector ROS for lipid peroxidation via enzymes LOX [Lipoxygenases] and POR [CytP450-OxidoReductases] and nonenzymatically by ferrous ions. #14: Enmeshed deposits of calcium + iron in peroxidized membrane lipids → cellular and organelle membrane rupture. #15: Caspase 3-induced apoptotic pathways: pyknosis + Karyorrhexis + karyolysis potentiated by primary and secondary ROS hydroxyl radicals (#13), calcium influx (#10), and ATP depletion. Apoptosis also releases substantial amounts of Damage-Associated Membrane Patterns [DAMPs]. DAMPs are also released by other ischemic processes. #15: DAMPs: the final common pathway for cell and organelle membrane rupture. #16: System xc^−^ receptor-mediated transport of Cystine is critical for production of the tripeptide Glutathione [GSH]. The lipid repair enzyme Glutathione Peroxidase [GPX4] catalytically utilizes GSH to inhibit lipid peroxidation. Abbreviations: TNF-r, Tumor Necrosis Factor receptor; Fas-r, Fas receptor; ETC, Electron Transport Chain; NaKA, Sodium–Potassium ATPase; ADP, Adenosine Diphosphate; ATP, Adenosine Triphosphate; M(i) Intact Mitochondria; M(f), Fragmented Mitochondria; N, Neutrophils; Gd, Gasdermin; ATPCal, ATP-dependent Calcium Export Pump; VoltC, Voltage-dependent calcium channels; Nu-k, Karyorrhexis of Nucleus; TF-r, Transferrin Receptor; 1-ROS, primary reactive oxygen species; 2-ROS, secondary reactive oxygen species; KC, Kupffer Cells; Sys-xc^−^, Cystine Transporter; GSH, Glutathione; GPX_4_, Glutathione Peroxidase; DAMPs, Damage-Associated Membrane Patterns; L, Normal lipids; L(p), peroxidized lipids; Fe^3+^, ferric ion; Fe^2+^, ferrous ion; Ca^2+^, calcium ion; OH, hydroxyl free radical; O_2_, oxygen Molecule.

The mechanisms of hepatic IRI have been widely investigated, but nevertheless remain largely unclear [10]. This is in part due to the number and complexity of the pathways implicated in these instances of cell death. It is therefore only possible to mention some of the processes which occur simultaneously to bring about cell death (Figure 1). Once ischemia is initiated due to either a lack of or limited blood supply, there is a resulting deficiency in oxygen, glucose, and other essential substances [11].

The lack of ATP production which ensues causes ATP-dependent ionic pumps to fail and transmembrane ion gradients to be lost. To attempt to maintain polarization, calcium release from the mitochondria into the cytoplasm is accompanied by a translocation of calcium ions from the extracellular to intracellular fluid. The formation of a mitochondrial permeability transition pore accompanied by the increase in calcium ion concentration leads to free radical production, cell shrinkage, and delayed cell death [12,13,14].

Hypoxia and IRI induce the expression of numerous cytokines with a range of effects: interleukin-1, tumor necrosis factor-alpha, etc. [11]. Many of these cytokines are produced by tissue macrophages. Their effects can range from increased nitric oxide production in the lungs and lung damage, in the case of tumor necrosis factor alpha (TNF-α), to rolling on the endothelial surface, in the case of IL-1α [4]. This exaggerated defense response culminates in systemic inflammatory response syndrome (SIRS). The dysregulated cytokine storm of SIRS is one of the factors which can lead to multi-system organ failure.

Following the reestablishment of blood flow, reactive oxygen species (ROS) are produced at an accelerated rate, which certainly contributes to reperfusion injury. There are numerous enzyme systems involved in the increase in ROS production following reperfusion: xanthine oxidase, NADPH oxidase, etc. [15,16]. The ROS produced by one enzyme can, in turn, activate and increase the rate of ROS production by another source. These ROS, namely hydrogen peroxide and hydroxyl radicals, initiate the peroxidation of lipid membranes, and the major consequences of this are the disruption of proper membrane permeability and, ultimately, cell death, sometimes by induction of apoptosis [15].

There are also more novel forms of cell death, such as ferroptosis, which have been linked to IRI-related damage. This form of cell death is characterized particularly by iron overload and iron-dependent lipid peroxidation. Increasing evidence is demonstrating the role of iron in ROS generation and oxidative stress build-up, especially with regard to GPX4 inactivation [17,18].

In summary, IRI represents a very complex array of interconnected pathological events and signaling pathways. The underlying mechanisms of IRI-related cell death in any organ system remain a topic of considerable research in which various models are actively at play [10,11,15]. The overload of calcium, upregulation of inflammatory reactions via cytokines, increased ROS production, and ferroptosis are just some of the pathways implicated in IRI-related tissue damage. As the mechanisms implicated in inflammation and organ failure continue to be updated and revised, this research may aid physicians in evaluating patient survival and initiating better interventions with more appropriate timing. This improved understanding will prove instrumental in preventing the worst outcomes of IRI.

## 3. Murine Model

Animal models are critical for understanding the mechanisms of IRI injury, as they allow for easy gain-of-function and loss-of-function genetic experimentation. While there are particular advantages to the use of any given animal model, the murine model has unique characteristics which have led to substantial interest in producing hepatic IRI in the livers of mice [19]. One of the major factors is the genetic malleability of the mouse system. The considerable utilization of genetically modified animals and the relative ease with which novel models can be generated to address particular questions have no match in the other animal models [19,20]. Indeed, transgenic knockout models, which are being more frequently used to study the molecular mechanisms of IRI, have far more significant use in mice than any other animal [21].

The relative ease of maintenance, low cost, and rapid reproduction rate of rodents also make them very advantageous for their use in these studies [22]. The increasing need for thoroughness in preclinical studies requires the use of additional animals, which is much more realistic when fewer resources are necessary to include a sufficient sample size.

While the use of the murine model has several advantages, there are numerous disadvantages to be considered as well, especially when considering the divergent aspects of the hepatic physiology of humans and mice. Certain larger animal models, such as the porcine model, can more closely resemble most aspects of human liver physiology. One such difference is the fact that rodents have lobated livers. Two aspects of lobular architecture that differ quantitatively between small and large mammals are the porto-central distance and the amount of connective tissue that is present in the portal tracts. Furthermore, liver lobules in small mammals like the mouse are more tortuous cylindrical structures than those in pig and human livers. Another difference is observed in the divergence in the bile acid pathways between humans and mice. Many studies analyzing cholestasis in mice have demonstrated the differences in the bile salt export pumps between the species [23]. Manipulation of the smaller liver also requires greater surgical skill and offers a smaller mass of tissue for freezing and later analysis of the extent of cell damage, such as PCR, immunohistochemistry, assay, etc. 

One study conducted by Yamada et al. utilized a murine hepatic IR injury model to study iron overload as a risk factor for liver damage. Four mice could be housed per cage and they had ad libitum access to food and water, requiring minimal management and oversight [24]. The mice were briefly anesthetized utilizing isoflurane and an atraumatic clip was placed across the portal vein, hepatic artery, and bile duct to interrupt blood flow exclusively to the left lateral and median lobes and removed after an hour to simulate reperfusion, a common practice [24,25]. After three hours of reperfusion, the mice were all sacrificed, and samples of the ischemic lobe and blood were collected. Cross-sectional analysis utilizing a sample size in the dozens allowed for statistical analysis of the serum parameters in the mice. This study is quite typical of those utilizing mice to study IRI.

## 4. Porcine Model

Compared to the murine model, the porcine model’s biliary systems are anatomically and functionally more similar to that of humans. Both pig and human livers consist of dexter, sinister, quadrate, and caudate lobes. Because of the more lobular shape of the pig liver, the dexter and sinister lobes are each divided into medial and lateral lobes. The only difference between them is the drainage of segment I or the caudate lobe of the liver, which is performed by the sinister hepatic duct in humans, while in swine it is drained by the dexter hepatic duct [26]. The caudate lobe is the autonomous zone of the liver, and its function is to receive blood from the portal venous and hepatic arterial branches in both human and swine livers. Furthermore, the swine’s venous system returns to the inferior vena cava, while the human venous system returns directly to the right atrium [26]. These advantages have prompted interest in producing hepatic IRI in swine liver. 

Due to restrictions and guidelines for the restraint and use of piglets, many labs choose not to acquire piglets and, therefore, are not able to utilize porcine models. Because of the procedural challenges associated with obtaining swine, there has been more limited research conducted on hepatic IRI in porcine models compared to murine models. Due to this, the model is primarily assessed based on its cost–benefit analysis and the anatomical and physiological similarities of swine to their human counterparts rather than on prior trials studying hepatic IRI.

The last few decades in particular have seen a rise in the use of porcine models, especially in studies investigating protective strategies by means of dynamic liver preservation, because the porcine liver is so anatomically and physiologically close to that of humans, with comparable organ dimensions and similar bile composition [27]. In a study by Maione et al., a porcine isolated liver perfusion model was found to adequately simulate the conditions of liver transplantation and provided a reproducible technique for studying early IRI events [27]. Their study also took note of the labor-intensive, technically challenging, financially burdensome nature of attempting to study liver transplantation in large animals [27]. They, however, held that isolated liver perfusion as a technique permits a reduction in the total number of experimental animals because a recipient is not necessary. Indeed, all steps preceding the transplantation of the graft can be replicated during isolated perfusion, mimicking the sequence of events of a liver transplant [27,28]. Therefore, isolated liver perfusion can be used to investigate the impact of warm/cold ischemia and protective strategies (i.e., drugs, dynamic preservation), since the reperfusion phase takes place in a controlled environment and offers an efficient alternative to the traditional liver transplant in large animal models such as this.

## 5. Methods of Ischemia

Reductions in sinusoidal diameter and blood flow are among the earliest changes which occur during reperfusion injury, and many procedures have been developed to closely mimic this process. Most animal models of IRI which have been developed involve complete vascular occlusion, low-flow ischemia, and/or segmented vascular occlusion [24,25]. In particular, complete ischemia by temporary vascular occlusion (with atraumatic vascular clamps) or permanent vascular occlusion (by ligation) in rodent models are currently the most commonly used surgical methods of inducing oxygen deprivation and intestinal, renal, or hepatic ischemic injury [25]. In most studies utilizing an animal model, heparin is also injected intravenously to prevent thrombus formation [25,29].

The liver receives a dual vascular inflow, providing approximately one-quarter of the total cardiac output, or a blood flow of 1500 mL/minute [30]. The portal vein provides 75% of the total hepatic blood flow, and the hepatic artery provides the other 25% [30,31]. The portal vein originates between the head and body of the pancreas at the confluence of the splenic and superior mesenteric veins and runs posterior to the bile duct and hepatic artery in the free edge of the lesser omentum. Now standard in hepatic surgery, hepatic pedicle clamping (Pringle’s maneuver) interrupts the arterial and portal venous inflow to the liver. For selective clamping of a lobe, however, ischemic margins can be created by complete devascularization by dividing the unilateral portal pedicle [32].

One issue of concern in all such procedures in animal models, due to the invasive nature of the surgery, is the need to manage the pain and suffering that occurs during and after the procedure; pain management practices must conform to the current and best guidelines. It is important to note, though, that experimental and clinical data have shown that anesthetics can have protective effects in several organs, including liver studies, that can mitigate IRI and have the potential to skew results [29]. This ‘anesthetic preconditioning’ has been well documented for decades, resulting from the use of sevoflurane, isoflurane, propofol, and other opioids [33,34,35,36].

## 6. Cell Lines

HepG2 is a human hepatocellular carcinoma cell line. It exhibits epithelial-like morphology and was isolated from a 15-year-old white male youth with liver cancer. This and other cell lines, such as Huh7, are frequently used to assess cell viability and gene expression under IRI-like conditions [37]. Although all such cell lines imperfectly mimic hepatocytes under physiological conditions, they still represent relevant models to rapidly screen for implications of target proteins toward the effective maintenance of intracellular structures because most of these mechanisms are well conserved in these cell lines.

There are a variety of components that may be utilized to simulate IRI. In a hepatic IRI study conducted by Emadali et al., an ischemia–reperfusion cell culture model was achieved by means of incubation in a cold storage solution treated with antimycin A (an inhibitor of cellular respiration) to induce a hypoxic condition [38]. The cold preservation solution was then replaced with warmed basal medium containing 10% fetal bovine serum [38]. In a similar study by Huang et al., a similar method was used to simulate IRI, but different samples were treated with different concentrations of ferrous or ferric iron along with a combination of iron chelators [34]. This was performed in quadruplicate as a means of assessing ferrous iron autoxidation in iron-loaded HepG2 cells. Trypan blue exclusion was utilized in both studies, though another method of cell viability testing could be made use of for statistical analysis [38,39].

Immortal cell lines are often used in research in place of primary cells. Although they often do not fully represent what is occurring in vivo, they offer several advantages, such as being cost-effective and easy to use, providing an unlimited supply of material, and bypassing ethical concerns associated with the use of animal and human tissue [37,40,41].

## 7. Normothermic Perfusion (NMP) Model

Several studies have evaluated the methods or agents used to mitigate IRI in liver transplants. However, most studies utilizing cell lines or murine models do not adequately recapitulate human IRI, limiting their translatability into clinical trials. Thus, a robust preclinical human therapeutic testing platform has been developed. There has been a growing interest in using normothermic machine perfusion (NMP), an organ preservation technology for liver support, which attempts to bypass cold ischemia time [42,43,44]. However, these pumps remain expensive and pose logistical challenges when they are transported to the recipient along with the donor liver. Moreover, they are unable to eliminate all forms of ischemia [45].

**Figure 2:**
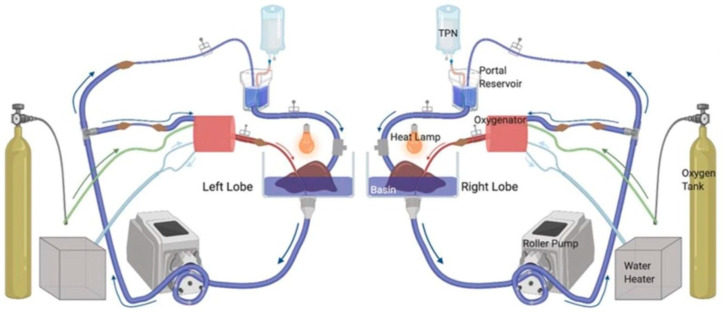
Schematic of machine perfusion model.

In order to advance strategies to expand the pool of viable livers available for transplant, the normothermic reperfusion model has been employed to investigate the mitigation of IRI in these types of livers. The NMP model is a unique human blood reperfusion system, in that it has dual arterial and portal blood supply channels. It allows two liver lobes from the same donor to be perfused concurrently, presenting an ideal internal control platform for the evaluation of therapeutic agents (Figure 2). Split human donor livers are placed on an extracorporeal membrane oxygenation machine. This model can pump arterial and portal blood separately and concurrently. One of the two lobes is treated and is assessed in comparison to the control lobe.

One example of how this system has been employed is in the testing of potential chelators for the treatment of ferroptosis. Ferroptosis is a recently described form of programmed cell death regulated by iron that has been implicated as a mechanism of pathogenesis in models of hepatic reperfusion injury [24,46]. In murine and cell culture models, the inhibition of ferroptosis has already been shown to be therapeutic, leading to a reduction in liver damage, lipid peroxidation, and inflammatory responses [47,48].

There are other types of machine perfusion, such as hypothermic machine perfusion (HMP). The main difference between these two methods is that NMP employs temperatures within the physiological range, while HMP employs temperatures lower than the physiological range. The function of HMP cooling the organ was developed to reduce the risk of graft failure, while NMP is effective at keeping the organ in a metabolically active state. HMP and NMP have demonstrated similar results in studies analyzing organ perfusion [49].

## 8. Conclusions

Hepatic ischemia–reperfusion injury is a potentially life-threatening process associated with significant morbidity and cell damage, and it is present in a wide range of liver surgeries. In order to properly elucidate the many mechanisms and pathways implicated in the pathogenesis of this cellular trauma, which is currently a major area of research, consideration of the models and methodologies that could potentially be at play is of great interest. From the perspective of cost, there is no doubt that cell line models are the most cost-effective, but their ability to perform modulation and clinical applicability is significantly limited, which has led to their increased use in the augmentation of existing results. The murine model, which is currently the most commonly used model to investigate hepatic IRI, is still inexpensive and has the most published data buttressing its reliability. Porcine models in general, though, are known for their suitability for studying human physiology. With regard to overall value, however, this team would argue that normothermic perfusion models are significantly underutilized in IRI research and offer a strong potential for therapeutic development; the direct translation into clinical studies vis-a-vis the utilization of human livers within a hyper-controlled setting, though costly in terms of the resources required, has shown promising results in the context of future applicability to liver transplant surgeries. A smaller sample size, which is inherent to porcine and machine perfusion models, may limit the generalizability of the results obtained from them. Nevertheless, this relative limitation also may yield entirely novel descriptions of gene modulation in livers. More efficient identification of the mechanisms at play in hepatic IRI may also contribute to an expansion of the pool of livers available for transplant.

## Data Availability

No new data were created or analyzed in this study. Data sharing is not applicable to this article.
